# A randomised clinical trial of a comprehensive exercise program for chronic whiplash: trial protocol

**DOI:** 10.1186/1471-2474-10-149

**Published:** 2009-12-02

**Authors:** Zoe A Michaleff, Chris G Maher, Gwendolen Jull, Jane Latimer, Luke B Connelly, Chung-Wei Christine Lin, Trudy Rebbeck, Michele Sterling

**Affiliations:** 1 The George Institute for International Health, The University of Sydney, George Street, Sydney, 2000, Australia; 2 National Health and Medical Research Council Centre of Clinical Research Excellence: Spinal Pain, Injury and Health, Division of Physiotherapy, The University of Queensland, Therapies Road, Brisbane, 4072, Australia; 3 Centre for National Research on Disability and Rehabilitation Medicine, The University of Queensland, Herston Road, Brisbane, 4006, Australia; 4 Australian Centre for Economic Research on Health, The University of Queensland, Herston Road, Brisbane, 4006, Australia; 5 School of Economics, The University of Queensland, Blair Drive, Brisbane, 4072, Australia; 6 Faculty of Health Sciences, The University of Sydney, East Street, Sydney, 2141, Australia

## Abstract

**Background:**

Whiplash is the most common injury following a motor vehicle accident. Approximately 60% of people suffer persistent pain and disability six months post injury. Two forms of exercise; specific motor relearning exercises and graded activity, have been found to be effective treatments for this condition. Although the effect sizes for these exercise programs, individually, are modest, pilot data suggest much larger effects on pain and disability are achieved when these two treatments are combined. The aim of this study is to investigate the effectiveness and cost-effectiveness of this comprehensive exercise approach for chronic whiplash.

**Methods/Design:**

A multicentre randomised controlled trial will be conducted. One hundred and seventy-six participants with chronic grade I to II whiplash will be recruited in Sydney and Brisbane, Australia. All participants will receive an educational booklet on whiplash and in addition, those randomised to the comprehensive exercise group (specific motor relearning and graded activity exercises) will receive 20 progressive and individually-tailored, 1 hour exercise sessions over a 12 week period (specific motor relearning exercises: 8 sessions over 4 weeks; graded activity: 12 sessions over 8 weeks). The primary outcome to be assessed is pain intensity. Other outcomes of interest include disability, health-related quality of life and health service utilisation. Outcomes will be measured at baseline, 14 weeks, 6 months and 12 months by an assessor who is blinded to the group allocation of the subjects. Recruitment is due to commence in late 2009.

**Discussion:**

The successful completion of this trial will provide evidence on the effectiveness and cost-effectiveness of a simple treatment for the management of chronic whiplash.

**Trial registration:**

ACTRN12609000825257

## Background

The most common injury following a motor vehicle accident is a whiplash injury to the neck [1]. This injury is of particular concern as approximately 60% of people experience persistent pain and disability 6 months after the original accident [2-4]. This group of people with chronic symptoms account for a disproportionately large percentage of the economic burden associated with whiplash injury [5,6]. In the state of New South Wales, Australia, in the period June 2007 to 2008 there were approximately 34 000 whiplash claims with an approximate total financial cost of $AUD 700 million [1]. In the United States the costs associated with whiplash are estimated to be of the order of $29 billion US dollars per annum [7].

Currently, there is a large number of treatments available to people suffering from whiplash symptoms including acupuncture, cervical collars, traction, exercise, massage, mobilisation techniques, electro-physical agents and the local application of heat or ice [8,9]. However, a recently published Cochrane review that evaluated conservative treatments for whiplash concluded that *no *clearly effective non-surgical treatments are currently available for chronic whiplash [8]. Studies of such treatments have reported zero, or small treatment effects; This result may be a "true negative", but it could also arise due to inadequate treatment dosage or the inability to target specifically the physical and psychological impairments associated with whiplash injuries [10].

There is growing evidence to support multimodal treatment strategies which combine exercise, manual therapy and psychological approaches. It has been shown that this type of treatment results in larger reductions in pain, greater patient satisfaction and a quicker return to work compared with conservative electro-physical treatments in individuals with acute whiplash [11]. In addition, the likelihood of recovery is increased when treatments are individually-tailored [12] to specifically target individual deficits and involve active rather than passive intervention strategies [8,13-15].

Over the last five years the chief investigators have been working to develop an effective treatment for chronic whiplash. The first developments were two exercise programs: graded activity [16] and specific exercise [17]. The programs were evaluated in separate randomised controlled trials with each trial demonstrating modest effects, with 10-20% of patients having a successful outcome, defined as the achievement of minimal or no pain and disability. In the investigators' opinion this success rate was too low to represent a solution to the problem of chronic whiplash and hence development of a more effective intervention was necessary.

Subsequently, a comprehensive exercise program was proposed which combined the two previously evaluated exercise programs. It was reasoned that rather than varying the training parameters of exercise intensity or duration (which may exacerbate people's whiplash symptoms), combining these two programs sequentially may result in greater improvements in pain and functional capacity.

The specific motor relearning component is designed to improve cervical and scapular muscle control, strength and endurance, coordination, kinaesthesia and balance [17]. Once any impairments in muscle control and co-ordination have been addressed, the strengthening and graded whole body exercise component is progressively introduced in order to improve participant's functional capabilities [16]. The proof of concept for the comprehensive exercise program was provided by a small unpublished pilot study which found that 56% of subjects reported minimal or no disability following this approach; which is a much higher success rate than the 10-20% success rate observed in the two earlier trials. However, robust evidence for the effectiveness of the new approach can only be provided by a larger controlled trial.

Therefore the aims of this study are to:

• Establish the effectiveness of a comprehensive exercise program for chronic whiplash (defined as whiplash symptoms for greater than 3 months' duration and less than 12 months' duration) as measured by reductions in pain and disability, and improvements in participants' impressions of overall recovery and quality of life.

• Conduct an economic evaluation of the exercise program.

• Investigate whether or not sensory hypersensitivity and symptoms of post-traumatic stress modify the effectiveness of the program.

## Methods/Design

### Funding

This investigator-initiated study has received funding from Australia's National Health and Medical Research Council, The New South Wales Motor Accident Authority (MAA) and the Queensland Motor Accident Insurance Commission (MAIC).

### Design

This multicentre randomised controlled trial will be conducted with recruitment sites in Sydney and Brisbane, Australia. Ethical approval has been gained from the University of Sydney (03-2009/11509) and the University of Queensland Human Research Ethics Committee (2008002059). Written informed consent will be obtained from all participants prior to their entry into the study.

### Study Population

**Figure 1:**
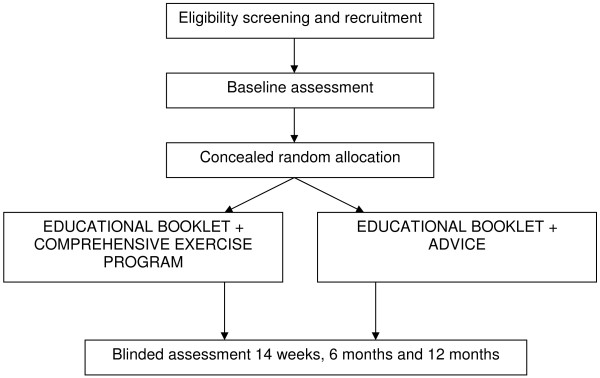
**Flow diagram of the trial**.

One hundred and seventy-two participants with chronic whiplash will be recruited (Figure 1 Flow diagram). Respondents will be recruited in Sydney and Brisbane via advertisements in local and metropolitan print media. In addition, the MAA and MAIC will assist with recruitment in Sydney and Brisbane respectively, by contacting potentially eligible claimants by post and inviting them to participate. The MAA and MAIC regulate the compulsory third party personal injury insurance scheme for motor vehicles registered in NSW and QLD.

Respondents will be screened via a telephone call to identify their eligibility. To be eligible for the trial participants must meet all of the following criteria:

### Inclusion criteria

• Grade I or II whiplash [6] of at least 3 months but less than 12 months duration (Table 1).

**Table 1:** Quebec Task Force clinical classification of whiplash

GRADE	CLINICAL PRESENTATION
**0**	No complaint about the neck. No physical sign(s).
**I**	Neck complaint of pain, stiffness or tenderness only. No physical sign(s).
**II**	Neck complaint AND musculoskeletal sign(s). Musculoskeletal signs include decreased range of motion and point tenderness.
**III**	Neck complaint AND neurological sign(s). Neurological signs include decreased or absent tendon reflexes, weakness and sensory deficits.
**IV**	Neck complaint AND fracture or dislocation.

• Currently experiencing at least moderate pain or moderate activity limitation due to pain (modified items 7 & 8 of SF36).

• Not currently receiving care for whiplash.

• Between 18 years and 65 years of age.

• Proficient in written and spoken English.

• Able to attend 4 assessment sessions at the trial centre.

### Exclusion criteria

• Known or suspected serious spinal pathology (e.g. metastatic disease of the spine).

• Nerve root compromise (Grade III whiplash).

• Confirmed fracture or dislocation at time of injury (Grade IV whiplash).

• Spinal surgery in the past 12 months.

• Any coexisting medical condition which would severely restrict participation in the exercise program e.g. traumatic brain injury, amputee.

• Any of the contraindications to exercise listed in the American College of Sports Medicine (ACSM) guideline [18] as assessed using the Physical Activity Readiness Questionnaire (PAR-Q).

### Randomisation

A computer-generated randomisation sequence, stratified for recruitment site (Sydney and Brisbane), will be produced prior to commencement of the trial by an independent researcher. Randomisation will occur immediately following the baseline assessment by opening the next sealed opaque envelope. Participants will be considered to have entered the study at the time that the envelope is opened.

### Treatments

All participants will be provided with the patient educational booklet 'Whiplash injury recovery: a self management guide' [19]. The booklet provides information about whiplash, advice on how to manage the symptoms of whiplash and outlines an exercise program to assist in reducing whiplash associated neck pain. During the twelve-week trial period all participants will be asked not to seek other treatments and where possible not to change current medications. In addition to the educational booklet participants will be randomised to receive either:

#### Advice session

Participants will receive one, thirty-minute consultation with a physiotherapist. During this consultation they will have the opportunity to read the educational booklet, have the exercises explained and any questions clarified. Participants will have the opportunity to contact the physiotherapist by phone on two occasions if they require any further clarification of the information contained in the booklet.

#### Comprehensive exercise program

**Figure 2:**
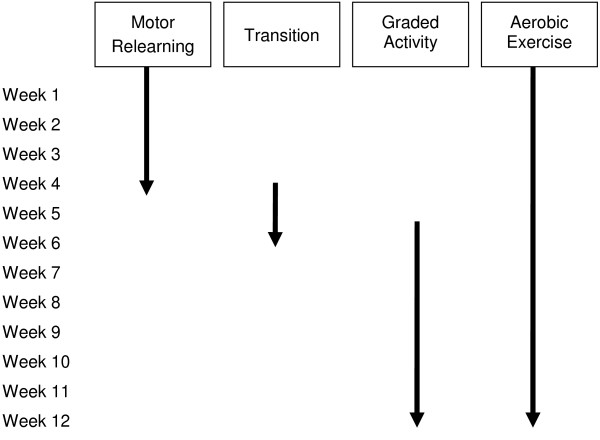
**Overview of the 12 week comprehensive exercise program**.

Participants will receive twenty, one-hour individually tailored and supervised exercise sessions over a 12 week period. Exercise sessions will be delivered at physiotherapy clinics by physiotherapists trained to deliver the comprehensive exercise program. Figure 2 provides an overview of the program.

The comprehensive exercise program begins with four weeks of specific cervical spine exercises, aerobic exercise and includes manual therapy techniques as required. Exercises include craniocervical flexion training, neck extensor training, scapular training, posture reeducation, sensorimotor exercises which include kinaesthetic sense, balance and eye movement control. This part of the program aims to train and improve the co-ordination and endurance capacity of the neck flexor, neck extensor and scapular muscles as well as address deficits in cervical spine joint position sense, eye movement control and balance as required [12].

The transition from the specific motor relearning program to the graded activity program occurs between weeks four to six and is dependent on the individual's progress through the earlier stage. During the transition period the focus is shifted from specific neck motor relearning exercises to integrate this control into functional whole body exercise. The graded activity program is intended to assist participants to achieve their progressively nominated functional goals. The individually-designed, sub-maximal program includes upper and lower limb muscle strength and endurance exercises, specific functional task practice (whole or part practice) as well as progressing the aerobic, neck flexor and neck extensor strength and endurance exercises from the earlier stage.

Participants will be provided with a home exercise diary which will outline their home exercise program across the 12 week period. The exercise program is to be completed on days they do not attend the treatment clinic and the exercise diary will be used to monitor participants compliance with the exercises.

Throughout the comprehensive exercise program, specific cognitive-behavioural therapy priciples will be used. These include: encouragement of skill acquisition by modelling, setting progressive goals, self-monitoring and positive reinforcement of progress [20]. By using these principles in conjunction with a progressive exercise program it is envisaged that participant's will progressively return to their pre-injury work and home activities, become more self reliant with the ability to problem solve and therefore be able to independently manage their condition and potential flare ups.

### Quality assurance

To ensure that the treatments are of a high standard and are delivered in accordance with the trial protocol, treating physiotherapists will attend a one-day workshop where they will be trained in the delivery of the treatment program and be provided with a therapist protocol and standardised recording documents. Each physiotherapist will have one treatment and one advice session audited by experts in the field. Furthermore, a one day workshop will be run midway throught the trial to ensure that good practice is maintained.

### Outcome assessments

Participants will be evaluated by an assessor who is unaware of group allocation at 14 weeks, 6 months and 12 months after randomisation.

The baseline assessment will involve the collection of the participant's demographic data including, information about their whiplash symptoms, accident history, medical history, current medications, previous investigations and treatment, employment and compensation status. The primary and secondary outcomes as well as potential effect modifiers will be measured at baseline and all other assessment time points.

The *primary outcome *is the average pain intensity over the last week as measured using a 0 to 10 point numerical rating scale [21]. *Secondary outcomes *are:

- Average pain intensity over the last 24 hours measured using a 0 to 10 numerical rating scale [21]

- Patient's global impression of recovery measured on a numerical rating scale from -5 (vastly worse) to +5 scale (completely recovered) [22].

- Perceived disability measured using the Neck Disability Index a 10-item questionnaire designed to measure neck specific disability [23] and the Whiplash Disability Questionnaire: 13-item whiplash-specific measure of disability [24].

- Health related quality of life measured using the SF-36 [25].

- Cervical range of movement using an inclinometer [26].

- Perceived function measured using the Patient-Specific Functional Scale: 3-item patient-generated measure of function [27].

Adverse effects of treatment will be identified using open-ended questioning at the 14 week follow-up assessment only. In addition to the primary and secondary outcomes, economic outcomes will be collected to assess the cost-effectiveness of the comprehensive exercise program. Details of the cost-effectiveness analysis will be reported in a separate manuscript.

#### Potential modifiers of treatment effect

Whiplash is a complex and heterogeneous condition and there is growing evidence to suggest that there is a subgroup of people who present soon after injury with altered sensory (mechanical and thermal) and psychological states; it is likely that these people are at an increased risk of developing persistent symptoms [28]. The following potential treatment effect modifiers will be measured in order to investigate whether they can predict those more likely to respond to treatment:

- Cold pain threshold will be measured over the cervical spine using a Thermotest System (Somedic AB, Sweden) [2].

- Pressure pain threshold will be measured over the cervical spine and the tibialis anterior using a Pressure Algometer (Somedic AB, Sweden) [2].

- Posttraumatic stress symptoms will be evaluated using the Impact of Events Scale [29] and the modified Post Traumatic Stress Diagnostic Scale [30]. Both are 15-item questionnaires used to assess posttraumatic stress symptoms that are related to a specific event.

- Signs and symptoms of catastrophising will be evaluated using the Pain Catastrophising Scale, a 13-item questionnaire developed to assess three components of catastrophising: rumination, magnification, and helplessness [31].

- The origin of participant's pain will be pain evaluated using the Self-report Leeds Assessment of Neuropathic Symptoms and Signs (S-LANSS) [32], a 7-item scale developed to identify neuropathic pain, as distinct from nociceptive pain.

### Data analysis

#### Effectiveness of the comprehensive exercise program

Intention to treat analyses will be used, exclusively, and will be conducted by an investigator who is blinded to group allocation. In the primary analysis the effect of treatment will be assessed separately for each outcome using linear mixed models with time as a repeated factor. The model will account for correlation over time within participants, correlation within clinics and potential confounders (e.g. important prognostic factors). Estimates of the effect of the intervention will be obtained by constructing linear contrasts to compare the mean change in outcome from baseline to each time point between the treatment and control groups, with adjustment for the other variables.

#### Potential modifiers of treatment effect

Effect modification will only be assessed for the primary outcome of average pain intensity over the last week. This will be done by including a predictor-by-treatment group-by-time interaction term to the mixed models analyses.

#### Sample size

This study has been designed to detect clinically important interaction effects for the potential treatment effect modifiers. There will be sufficient power to detect the following effects: 2 units on the 0-10 pain intensity scale (estimate for SD = 2.00), 1.5 units on the 0-10 Patient Specific Functional Scale (estimate for SD = 1.5), 30 units on the 0-130 Whiplash Disability Questionnaire (estimate for SD = 30), 2.0 units on the -5 to +5 Global impression of recovery scale (estimate for SD 2.0), 6 units on the 0-50 Neck Disability Index (estimate for SD = 5.6), 15 points on the 0-100 SF36 physical summary score (estimate for SD = 10.0) and 15 points on the 0-100 SF36 mental summary score (estimate for SD = 11.0). However, the study is powered to detect main effects of half this size.

Standard deviation estimates have been taken from previous trials that recruited a similar patient cohort [16,17,24]. With specifications of alpha = 0.05, power = 0.80 and allowing for up to 10% loss to follow-up and 10% treatment non-compliance, a sample size of 86 participants per group will allow us to detect an interaction effect size equal to 1.0 times the SD as specified above and a treatment main effect of 0.5 SD.

#### Data integrity

The integrity of trial data will be monitored by regularly scrutinising data sheets for omissions and errors. Data will be double entered and the source of any inconsistencies will be explored and resolved.

## Discussion

### Controlling bias

This trial includes a number of key methodological features which will minimise bias including randomisation, concealed allocation, blinded outcome assessment and intention to treat analysis. Due to the nature of the intervention we are unable to blind therapists or participants.

### Outcomes

The outcomes that have been chosen are consistent with published guidelines for the management of whiplash associated disorders [15,33]. The combination of generic pain and disability questionnaires, condition-specific questionnaires and patient-generated measures of disability will provide a comprehensive overview of the participants' clinical condition and health-related quality of life.

### Timeline

Recruitment of participants will commence at the end of 2009 with all participants' recruited and treatment completed by December 2010. Twelve-month follow-up data will be completed by December 2011 with data analysis and manuscript completed by mid 2012.

## Conclusion

This study will provide a definitive evaluation of the effectiveness and cost-effectiveness of a comprehensive exercise program for chronic whiplash. The evaluation of this treatment is critical because current treatments available for people with chronic whiplash only offer at best, modest benefits.

## Competing interests

The authors declare that they have no competing interests.

## Authors' contributions

ZAM, CGM, GJ, JL, LBC, CWCL, TR and MS were responsible for the design of the study. CGM, MS, GJ, JL and LBC procured funding. All authors have read and approved the final manuscript.

## Pre-publication history

The pre-publication history for this paper can be accessed here:

http://www.biomedcentral.com/1471-2474/10/149/prepub
